# EXAMINING THE ASSOCIATION BETWEEN LAND COVER AND PHYSICAL MOBILITY: FINDINGS FROM THE NHATS STUDY (2021)

**DOI:** 10.1093/geroni/igad104.3318

**Published:** 2023-12-21

**Authors:** Rohini Perera, Carina Gronlund, Grace Noppert, Kate Duchowny

**Affiliations:** University of Michigan, Ann Arbor, Michigan, United States; University of Michigan, Ann Arbor, Michigan, United States; University of Michigan, Ann Arbor, Michigan, United States; University of Michigan, Ann Arbor, Michigan, United States

## Abstract

The vast majority (88%) of adults wish to age in place. An aesthetically pleasing environment has been found to be associated with better physical functioning and reduced disability, however specific land cover features (i.e. blue, green, urban spaces) and their relation to mobility have not been fully investigated. Data come from the National Health and Aging Trends Study (NHATS, 2021), a nationally representative sample of Medicare beneficiaries, and the National Neighborhood Data Archive (NaNDA), a data repository with census-tract measures of land cover. Mobility was assessed using the objective Short Physical Performance Battery (SPPB) and subjective physical capacity (PC) (range = 0-3 for both measures). Higher categories for both measures indicate better mobility. The analytic sample included 2,939 individuals (58.3% women) with mean age 81.8 years (SD = 6.4) from 2,071 census tracts. In fully-adjusted ordinal logistic regression models, compared to those in the lowest SPPB category, each percentage increase of blue space was associated with a 3.6 greater odds of being in the next highest category of SPPB (aOR= 3.61, 95% CI = 1.20-10.84 ). In contrast, each percentage increase of urban space was associated with a 18% decreased odds of being in the next highest category of SPPB (aOR= 0.82, 95% CI = 0.67-0.99). Similar associations were observed with PC, although were not statistically significant. Since decreased mobility is associated with numerous negative health outcomes, results from this study highlight the need to consider land cover as a potential policy lever to promote mobility among older adults.

